# Case Report: An integrative approach to psychotherapy for the mother of a daughter with bulimia nervosa

**DOI:** 10.3389/fpsyt.2025.1595758

**Published:** 2025-08-19

**Authors:** Qiu Yuan Zhang, Hong Yi Wang, Yan Hong Liu, Hui Yan

**Affiliations:** ^1^ Education and Science Department, Jiangsu Second Normal University, Nanjing, China; ^2^ Humanistic Management Department, Hebei Chinese Medicine University, Shijiazhuang, China; ^3^ JiaoJiang Psychosomatic Department, Taizhou Second People Hospital, Taizhou, China

**Keywords:** eating disorders, caregivers, integrative therapy, psychological care, mental health management

## Abstract

Caregivers is a crucial factor in recovery and psychological intervention of individuals with eating disorders which are often-overlooked. This study explores the role of integrative therapy in promoting the psychological development of the mother of a daughter with bulimia nervosa, improving family structure, and facilitating the patient’s recovery. A 43-month, 54-session intervention was conducted with the mother of a woman with eating disorder using an integrative therapy approach primarily based on Bowen family systems therapy, drawing, and narrative therapy. The mother demonstrated improvements in emotional expressiveness, psychological distress, caregiving burden, and family structure, while her daughter experienced a reduction in eating disorder symptoms, improvement in the mother-daughter relationship, and restoration of social functioning, enabling the establishment of personal intimate relationships. In this context, the therapeutic value of integrative therapy deserves further attention. Meanwhile, the issue of mental health management is also reflected in this paper.

## 1 Introduction

“Eating disorders” (ED) refers to a group of syndromes characterized by abnormal eating behaviors and psychological disturbances, often leading to significant changes in body weight and physiological dysfunction. The primary types include anorexia nervosa (AN), bulimia nervosa (BN), and binge eating disorder. ED are most prevalent among adolescents aged 12–25 years, with a higher incidence in females (1). Beyond core symptoms such as food restriction, excessive exercise, self-induced vomiting, and binge eating, ED are often accompanied by severe psychiatric and physical complications, including hypoglycemia, hypokalemia, and liver or kidney dysfunction, that can have serious, life-threatening consequences. Individuals with ED also face increased risks of physical health issues, suicidal ideation, treatment dropout, poor prognosis, and relapse (2). Caregivers of patients with ED often exhibit heightened emotional expressiveness and excessive criticism, which can reinforce ED symptoms and exacerbate the caregiver’s psychological distress and burden, ultimately perpetuating a vicious cycle (3). In this phenomenon, known as “conformity and empowerment,” a caregiver’s attempts to cope with their own stress inadvertently reinforce the patient’s ED symptoms and maladaptive behaviors. ED patients often struggle with emotional regulation difficulties, which include poor emotional awareness and understanding, a lack of effective coping strategies, avoidance behaviors in pursuit of idealized goals, and an inability to engage in goal-directed actions during distress (3).

The development and persistence of ED are closely linked to dysfunctional family relationships, particularly in patients with AN, who often experience “symbiotic” or “enmeshed” family dynamics (4). Additionally, the conflict between independence and traditional Chinese family norms has been identified as a key factor in both the onset and maintenance of ED, highlighting the importance of incorporating caregivers into the treatment framework (5). Therefore, the provision of illness-related education, treatment information, and opportunities for caregivers to participate in psychological interventions can help break the disorder cycle and enhance the outcomes of recovery for patients with ED.

This study employs an integrative approach incorporating Bowen family systems therapy, drawing therapy, and narrative therapy to explore the process of psychotherapy of a mother caring for her daughter with BN. Through an analysis of 54 therapy sessions conducted over a 43-month period, the study illustrates the role of psychotherapy in alleviating caregiver distress, addressing systemic family issues, and ultimately contributing to improvements in the patient’s symptoms. This integrative approach led to positive adjustments in the mother’s emotional expressiveness, psychological distress, caregiving burden, and family structure. Concurrently, the patient’s eating disorder symptoms gradually subsided, her social functioning improved, and she was able to establish intimate relationships and embark on a professional career.

The integrative approach, which has been developed over more than 100 years, is an old and vibrant theme and an important movement in psychotherapy and psychological counseling. Psychotherapy integration has four main types: theoretical integration, technical eclecticism, common factors, and assimilative integration (6). Psychotherapy integration has undergone a brief but complex historical development. The causal interpretation provided by any single theoretical school cannot provide a comprehensive, systematic understanding of the problem because the organism and its environment are subject to a two-way interaction. The systematic integration of the various schools of thought can thus aid in revealing the nature of the problem and choosing appropriate counseling strategies (7). Fernández-Álvarez Héctor has explored the practical reasons for integrative psychotherapy: Over time, many notionally purely formal approaches have incorporated strategies, interventions, and techniques from other purely formal psychotherapies, which can be considered a de facto integration at both the practical and theoretical levels (8).

This study will discuss the key points and significance of psychological intervention for caregivers of patients with BN from the perspective of mental illness management and psychological nursing. At the same time, the clinical value in practice of the integrative approach to psychotherapy is discussed.

## 2 Case description

### 2.1 Client information

Ms. D is a mild-age woman who has been married and has a daughter. D was born after the founding of the new China, which was marked by three years of natural disasters and significant social and political turmoil. She was raised in a family where her father passed away early, and her mother currently lives in their hometown with her elder sister and brother. Within the family, her eldest sister acts as the decision-maker, her second sister is highly critical, and her brother lacks a sense of responsibility. During her childhood, Ms. D’s father doted on her. However, due to her ordinary appearance and modest family background, she was belittled by her teachers at school. Despite this, she found warmth and support at home. Upon entering university, Ms. D excelled academically, gained recognition from her teachers, and began receiving admiration from her male classmates, which boosted her self-confidence. After completing her postgraduate degree, she worked in a government office but struggled to adapt, feeling inadequate in interpersonal relationships. Shortly after her daughter’s birth, she failed the civil service entrance exam, leaving her deeply frustrated. Ms. D mentioned that when her daughter was 1 to 3 months old, she experienced a significant shock to her physical and mental well-being that caused her to lack breast milk, making it difficult to feed her daughter. In her work, she was at an early stage of her career with unstable development and the future felt very uncertain, which caused her extreme anxiety. Consequently, she failed the civil service entrance exam again.

Ms. D had a close relationship with her husband during her daughter’s childhood, and the couple often went out together, frequently leaving their daughter alone at home. Her husband took on more responsibility for tutoring and caring for their daughter, while Ms. D primarily set high expectations for her. From elementary school onward, her daughter boarded at school and eagerly anticipated her mother’s visits, hoping to be picked up and brought home each week. However, Ms. D often praised other children in front of her daughter, making her deeply uncomfortable. Although the daughter was emotionally dependent on both parents, her emotional needs were never fully met.

After failing the civil service entrance exam, Ms. D faced significant limitations in her career development and demonstrated inadequate competence for her job. At the same time, she experienced difficulties in interpersonal relationships. Ms. D thus left her government job around the time her daughter was in the fifth grade of primary school and experienced depression and significant weight gain. During this time, she also discovered that her husband was having an affair, which led to their emotional estrangement. Approximately a year after leaving her job, Ms. D started her own studio, whose income was quite good. Ms. D gradually regained her confidence, yet her relationships with both her husband and daughter remained distant. Throughout her daughter’s middle school years, Ms. D experienced ongoing feelings of depression but did not seek medical treatment. During this time, she was provided with great care and support by her daughter, giving her feelings of emotional warmth.

After her daughter completed her college entrance exams, Ms. D encouraged her daughter to join her in losing weight, during which the daughter developed mild bulimic symptoms. During the first year in the university, her daughter’s symptoms worsened. At the end of her freshman year, she was diagnosed with BN, which led to a leave of absence for treatment.

### 2.2 Reason for seeking therapy

In the fall of 2018, Ms. D’s daughter was diagnosed with BN at a top-tier hospital in Changjiang Triangle Area, where she began receiving conventional medication and psychotherapy, and she took a semester off from school. In March 2019, she returned university to continue her studies. However, in January 2020, she relapsed and took another leave of absence. In March 2020, she participated in an outpatient eating disorder rehabilitation program at a psychiatric hospital, consisting of patient and caregiver parts. In the patient part, Ms. D’s daughter underwent DBT treatment and individual psychotherapy; in the caregiver part, Ms. D received psychological education and a follow-up of her daughter’s treatment, and underwent individual psychotherapy. In May 2020, Ms. D began her own psychotherapy following the recommendation of the rehabilitation program physician.

The therapist is a member of the project team, holds a doctoral degree in applied psychology, and is a registered psychologist. During her doctoral studies, she received training in analytical psychology (including sandplay therapy), psychoanalysis, and existential-humanistic oriented therapy. After her doctorate, she began working in a psychiatric hospital, conducting psychotherapy, and completing her clinical training in psychological treatment. During her clinical training, the therapeutic technique training focused on CBT, family therapy, arts therapy (primarily drawing techniques and psychodrama), narrative therapy, and mindfulness. Now, the therapist works as a lecture in the major of applied psychology in a university and also a part-time therapist in hospital and community organizations.

### 2.3 Initial impressions

Ms. D is well-proportioned appearance and wears simple, neutral-colored cotton-linen clothing. She has short hair that exposes her forehead and ears and wears simple, neutral-colored cotton-linen clothing. She speaks fluent Mandarin and conveys a sense of urgency. Although her eye contact is somewhat evasive and she demonstrates moderate anxiety, she is relatively open about herself and is willing to seek help. However, she shows some avoidance when confronting her sense of powerlessness. She is willing to discuss her daughter’s eating disorder medication and treatment, as well as the practical challenges in university life those her daughter faces. She displays a good level of trust in the therapist.

## 3 Psychological assessment

During the initial session, Ms. D completed a series of psychological assessments with the following results: PHQ-9^1^ (Depression Scale): 9, GAD-7^2^ (Anxiety Scale): 13, PDQ-4^3^ (Personality Diagnostic Questionnaire): Paranoid Traits: 4.34, Narcissistic Traits: 5.87, Dependent Traits: 5.35, Passive-Aggressive Traits: 4.77. The results suggest that Ms. D was experiencing mild depression and moderate anxiety. She exhibited paranoid and narcissistic tendencies, as well as a strong dependency in relationships and passive-aggressive behaviors.

From a biopsychosocial perspective, Ms. D’s psychological characteristics are closely linked to her upbringing, sociocultural background, and family environment. Her core self-development and emotional stability remain somewhat fragile.

## 4 Therapy process

### 4.1 Therapy timeline

See **Table 1**.

**Table 1:** Therapy timeline.

Sessions	Session 1-27			Session 28-39	Session 40-44		Session 45-50	Session 51-54
Frequency	1session/2w			1session/3w	1session/4w		1session/6w	1session/8w
Stage	Problem presentation		Problem transformation	Function Restoration	Social development			Termination and Separation
	Session 1-12		Session 13-27	Session 28-39	Session 40-50			Session 51-54
Clinical events	① Expression of psychological conflicts ② Recognized the lack of empathy, rejection, and high expectations toward her daughter ③ Recognized her own personality traits, behavior patterns, and parenting style ④ Recognized that her anxiety served as the emotional core of the family system ⑤ Re-examination of her relationship with her husband		① Identifying and addressing family triangulation patterns ② Breaking the cycle of transferring anxiety, anger, and control to her daughter ③ Differentiation from her daughter (differentiation of self) ④ Marital relationship improved	① Regulating attitudes and responses toward her daughter’s eating issues ② Exploring and understanding her daughter’s personality traits ③ Balancing personal career and family life	① Develop a secure attachment ② Monitor the client’s daughter’s binge eating symptoms in the therapeutic alliance ③ Feel release about daughter’s eating habits ④ More independence			① Respect the daughter’s personal choices and autonomy ② Reduce workload, allowing herself more time for quiet reflection
Symptom evolution	① Development of self-awareness ② Development of self-reflection		① Awareness of underdeveloped mentalization abilities ② Willingness to change ③ “I have finally become a mother” (Ms. D’s most profound realizations) ④ The client began to take responsibility for preparing family meals ⑤ The client’s husband increased the time spent at home	① Daughter’s binge eating episodes decreased ② Prepared herself for the possibility that her daughter’s binge eating symptoms might never completed disappear	① BN daughter was nearing the completion of her postgraduate studies and was preparing to get engaged to her boyfriend. ② Discussing plans for her later years with the therapist ③ Contribution to the rehabilitation of individuals with mental health disorders ④ Maintaining a healthy family dynamic			① ① Daughter had completed the internship and secured employment in the same city as her fiancé. ② Client rekindled intimacy with her husband ③ Client stopped being critical of her siblings ④ Client accepted that her daughter might not always respond to message
Length	75–100 mins			75–100 mins	75 mins			60 mins
Psychotherapy Schools	Bowen family systems therapy							
	Drawing 16 Theme: our family			Narrative therapy 32, 36, 47				
				Session 32 assigned topic: my daughter Session 36 assigned topic: gluttonous girls Session 47 assigned topic: us in ten years				
Face-to-face session	Session 1-3	Session 15, session 22				Session 42, session 50		Session 54

### 4.2 Therapy process analysis

#### 4.2.1 Therapy assignment

The client’s therapy is a component of her daughter’s eating disorder rehabilitation program. In the treatment program, individual therapy for family members is supplemented by one-on-one supervision. The supervisor designed Ms. D’s individual therapy plan after understanding her daughter’s growth, illness, and treatment experiences and discussing them with the therapist.

This treatment primarily revolves around family therapy, and drawing techniques and externalization and rewriting techniques from narrative therapy serve as supplementary methods for more effective results. The design of this treatment plan is based on the hypothetical components of family therapy; drawing on Bowen family systems therapy, it can be hypothesized that the patient’s symptoms are related to the emotional system of the core family, which mainly comprises the family’s triangulated relationships and the parental projection processes. Parents may experience issues related to self-differentiation, intergenerational transmission, and sibling positioning within this dynamic. Many of these issues operate at an unconscious level, particularly because the patient’s mother exhibits noticeable emotional problems. While the therapist can explain and interpret these processes from her own perspective for the client to some extent, to help the client achieve insight during the therapy process, more intuitive methods are needed to assist her in making the unconscious conscious. In this regard, drawing techniques and externalization techniques from narrative therapy have particular advantages. Therefore, in the first phase of Ms. D’s individual therapy, the therapist appropriately introduced drawing techniques and narrative therapy as the therapeutic relationship stabilized.

Before the treatment commenced, the client stated that the proposed weekly session frequency was too tight and requested longer biweekly sessions to avoid anxiety due to time constraints. Considering the client’s age and treatment goals, the treatment schedule was designed to be flexible, with the session lengths set at 60–100 minutes and adjusted as per the client’s development.

#### 4.2.2 Integrative psychotherapy process

##### 4.2.2.1 Stage one (problem presentation, sessions 1–12)

During the first stage, the client primarily revealed conflicts in her relationship with her daughter and her difficulty in accepting her daughter’s illness and personality, and conveyed her inner struggles regarding these issues. As the therapeutic alliance stabilized and deepened, the therapist appropriately engaged in confrontation to help the client recognize her lack of empathy, rejection, and high expectations toward her daughter, and the impact of her personality traits, behavior patterns, and parenting style on her daughter’s illness. During this stage, the client acknowledged her poor self-reflective abilities and limited understanding of others and her environment—psychological characteristics that disrupted the formation of a secure attachment between herself and her daughter.

A strong therapeutic alliance facilitated the development of self-exploration and self-awareness. Ms. D gradually came to recognize that her anxiety served as the emotional core of the family system, influencing the relationships among the family members. Her daughter consistently played the role of emotional problem-solver for the mother within the family system. This stage also guided the client to reexamine her relationship with her husband, highlighting how excessive self-focus contributed to her emotional absence in the marriage and the emotional distance between them.

Low self-reflective capacity is a psychological defense that clients use to cope with early trauma. Her difficult family circumstances during childhood and her devaluation by her teachers have led to a crippling sense of inferiority and long-term suppression, anger, and fear. After establishing her own family, the client’s daughter, as the most vulnerable part of the core family, became a channel for the client to release her emotions. In a safe therapeutic relationship, the client can reflect on and express her emotions to the therapist. Using containing and mirroring, the therapist guided the client in deep self-exploration and self-awareness.

##### 4.2.2.2 Stage two (problem transformation, sessions 13–27)

By this stage, the client had become aware of her underdeveloped mentalization abilities and demonstrated a willingness to change. The primary focus of this stage was to help the client redefine her maternal identity and understand family relationships while adjusting the family dynamics. The therapist guided the client in identifying and addressing triangulation patterns within the family, helping her break the cycle of transferring anxiety, anger, and control onto her daughter due to unresolved conflicts with her husband. The client’s differentiation from her daughter and development of a separate self-marked a key milestone. One of the client’s most profound realizations during this stage was: “I have finally become a mother.” This progress can be explained by the view of differentiation of self (DoS) of Bowen Family System Theory, which means that the individual is capable of maintaining emotional objectivity amid high levels of anxiety in a system involving the partner, children, siblings, and friends. Bowen hypothesized that DoS is a stable trait that emerges in early adulthood, although it can be disrupted by significant stressful life events, prolonged stressful situations, or psychotherapy. Individuals with higher levels of DoS are good at modulating emotional arousal during challenging interpersonal situations and show greater emotional maturity and interpersonal competence (9).

Additionally, the client’s marital relationship improved, as evidenced by her taking responsibility for preparing family meals and her husband spending more time at home. While she still found interactions with her daughter challenging, she no longer resorted to avoidance or criticism. Instead, she tried to overcome her psychological discomfort, explored appropriate ways to accompany her daughter, and experienced a deeper emotional connection with her. These moments of connection brought joy to the client.

**Figure 1:**
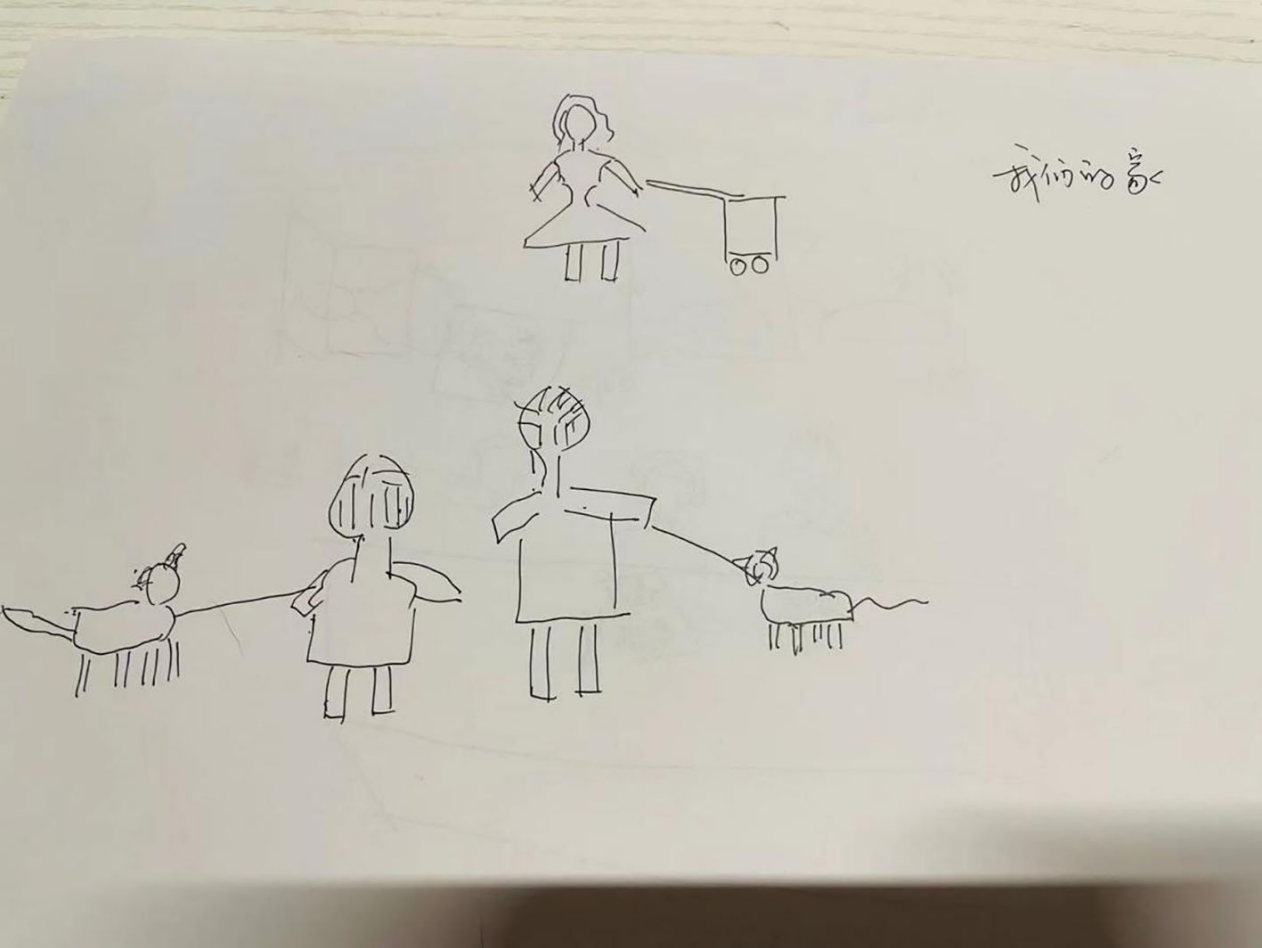
Our family (drawn by Ms. D).

**Figure 2:**
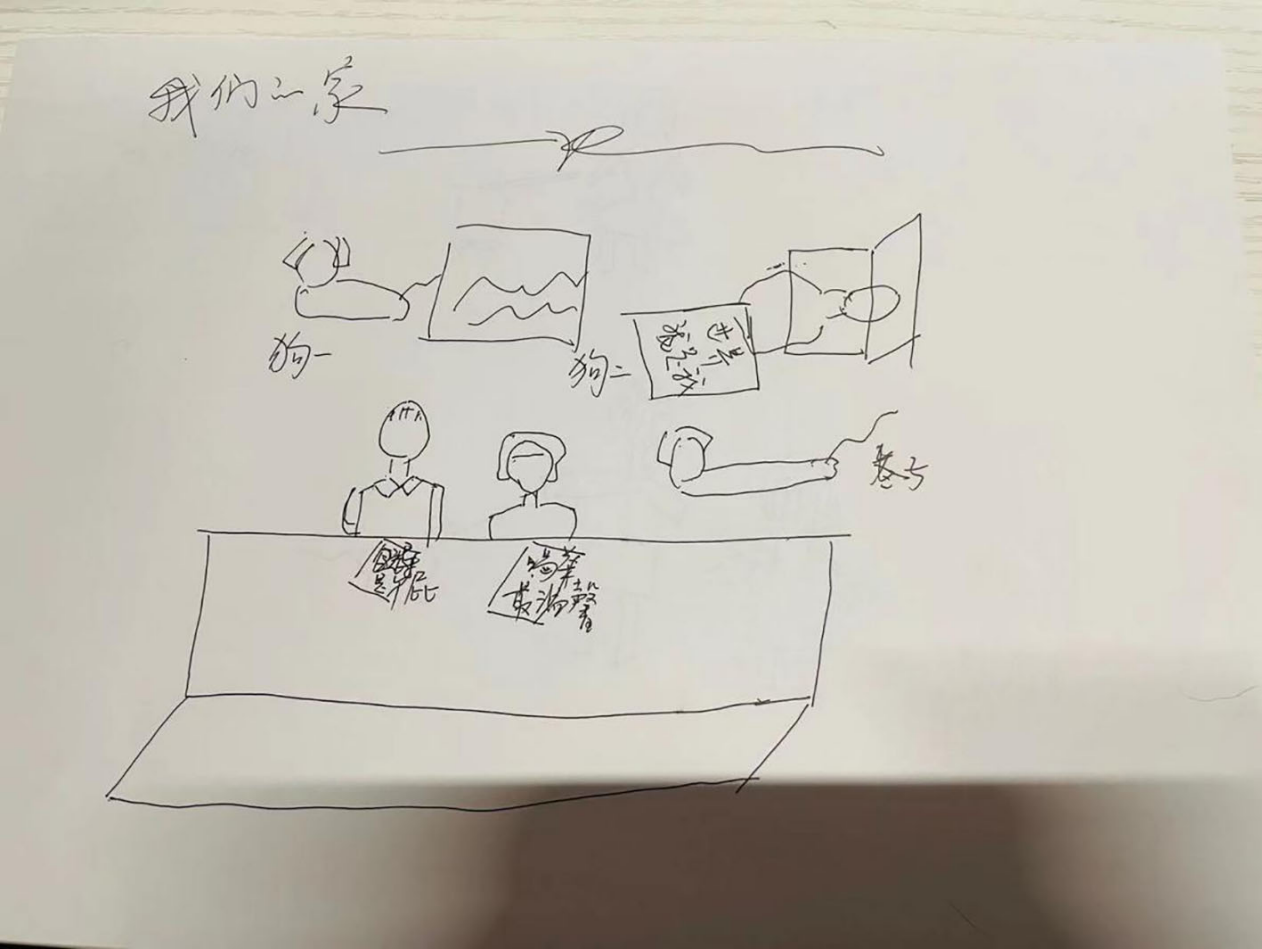
Our family (described by Ms. D’s husband and drawn by Ms. D).

In Session 16, drawing techniques were introduced, with the client and her husband collaboratively completing the themed drawing “Our Family” (**Figures 1**, **2**). The daughter did not participate in this activity.

The people in front are Ms. D and her husband, and the girl at the back is her daughter. The couple each holds a dog, which are the two pet dogs in the family.

The people in front are the father and Ms. D. The words in front of the father are “leaders are rubbish,” and those in front of Ms. D are “drinking tea is the warmest and sweetest.” Behind them are two dogs and the father lying down. The words before him are “I am the world.” An alleyway lies between the front and back. Two pet dogs are behind the alley. There is no daughter in the picture.

Art techniques mainly employ nonverbal symbolic means to express the hidden contents in the unconscious (10). Drawing is generally based on the psychological principles of projection and brain lateralization, as it helps patient clients divert their attention and express their inner feelings through creative activities, thus relieving physical and mental symptoms (11). In addition, drawing releases unconscious information that may be used to understand the impact of the visitor’s past motives, views, and experiences on the present and make appropriate adjustments to help the visitor better understand himself and his connection with the external world (11).

In Session 16, a structured drawing assignment was introduced, which visually depicted the characteristics of the family’s structure and relational dynamics. The client and her husband formed a close alliance, whereas the daughter’s connection to them was weak. In the client’s drawing, the daughter was depicted as separate from her parents, whereas in her husband’s drawing, the daughter was absent altogether. This suggests that while the daughter’s relationship with her parents existed on a cognitive level, there was emotional rejection by both parents. The fact that the daughter refused to participate in the client’s (the mother) homework also indicates that she does not agree with the union of the family. This can be interpreted as: there is a factual connection between the parents and the daughter, but there is a lack of psychological attachment. At the same time, it also reflects the conflicts within the family’s dynamic system and the emotional inconsistency among family members.

Consequently, the daughter lacked emotional support and nurturing during her upbringing, and EDs are often linked to emotional deprivation. By providing this feedback to the client, the therapist guided her to reflect on adjustments to the family structure and her emotional investment in her daughter.

##### 4.2.2.3 Stage three (function restoration, sessions 28–39)

As the client’s issues transformed, positive trends emerged in her relationships with her daughter and husband, as well as in her daughter’s recovery from her eating disorder. Notably, the client gradually relinquished control over her daughter’s eating habits. She also steeled herself for the possibility that her daughter’s binge-eating symptoms might never completely disappear; she expressed a willingness to accept this and continue providing her daughter with psychological support.

The therapeutic work in this stage mainly included the following: 1) Regulating attitudes and responses toward her daughter’s eating issues and tracking her daughter’s follow-up psychiatric visits. 2) Exploring and analyzing her daughter’s personality traits. 3) Balancing her personal career and family life. As the frequency of her daughter’s binge-eating episodes decreased, the client began planning for her daughter’s post-graduation life, exploring ways to foster her independence. She considered renting an apartment for her daughter to help her develop skills for independent living and started preparing for decisions about whether her daughter would pursue further studies or begin to work. During this stage, the client felt a growing sense of mutual support within her family. She expressed that she had not only regained her role as a mother but also reestablished herself as a wife and began to articulate a sense of happiness.

Ekinci and Tokkaş pointed out that although there is currently no set of recognized or relatively fixed technical procedures for narrative therapy, the main steps of narrative therapy for clinicians in treatment are: externalization of the problem, searching for a unique outcome, building a new story, and enriching the new story (12). One of the important theoretical foundations of narrative therapy is social constructivism, which sees problems as existing in language and thus changeable by constructing language.

At the end of Session 31, the therapist assigned Ms. D a narrative therapy homework task titled “My Daughter.” This exercise primarily used the technique of externalizing the problem by encouraging the client to objectively describe her daughter, thus clarifying the issues in their relationship. In her written response, the client only expressed appreciation and fondness for her daughter’s appearance, personality, and behavior. However, in the following session (Session 32), the client, almost shouting, declared, “No, I don’t actually like her behaviors! I don’t like her!” For the first time, she vented her rejection, resentment, and struggle with accepting her daughter. This session marked a critical turning point in the therapeutic process. Following this realization, the client stopped trying to convince herself that she fully accepted and admired her daughter. Instead, she began acknowledging the difference between her real daughter and her idealized image of her, respecting both her daughter’s authenticity as an independent individual and her own feelings of non-acceptance. However, she expressed a commitment to continue listening to her daughter and to remain present and responsive whenever she needed her. This shift demonstrated the effectiveness of externalizing the problem in narrative therapy.

After Session 35, the therapist assigned a second narrative therapy homework task titled “Gluttonous Girls.” Through her daughter’s treatment program, the client had the opportunity to engage with other families of individuals with EDs, allowing her to learn about young women aged 10 to 27 who shared similar struggles. Through interactions with other parents, she realized that her daughter’s binge-eating behavior was driven by unmet emotional needs, which had been present since infancy. Furthermore, she recognized that dysfunctional family structures were a common factor among individuals with ED. This narrative therapy homework incorporated the techniques for identifying unique outcomes and constructing a new narrative. By doing so, the client reframed her daughter’s binge-eating behavior, understanding it as an emotional coping mechanism rather than just a problem to be solved. She also learned to separate her daughter’s identity from her eating disorder and, through narrative language, envision a healthier future for her daughter. This shift encouraged the client to recognize and appreciate her daughter’s strengths beyond her eating disorder.

In the narrative work of this counseling, the main technique was “problem externalization” in narrative therapy. Ms. D’s expression was all about her appreciation of her daughter, but in the counseling conversation, she directly expressed her resistance, lack of appreciation, and inability to accept her daughter. The 32nd counseling session was an important turning point. After this counseling session, she was no longer convinced or implied to herself that she appreciated and accepted her daughter; she began to accept the difference between the reality of her daughter and the ideal daughter within herself, and respected the authenticity of her daughter as an independent individual and her lack of acceptance of her daughter. However, she said she would continue to listen to her daughter and remain present and responsive when she needed her. This is the utility of problem externalization in narrative therapy.

As Ms. D’s DoS gradually progressed, she became more flexible in regulating her own anxiety, fear, and anger. She deeply recognized that she and her daughter are two independent individuals, as are she and her husband. As a result, Ms. D began to understand others from their own perspectives and demonstrated more respect and tolerance in her interpersonal relationships. Reflecting on her past behaviors, she recognized many of her own shortcomings and could see her own transformation.

##### 4.2.2.4 Stage four: social development (sessions 40–50)

During this stage, the frequency of the sessions was gradually adjusted to once every 4–6 weeks. At this stage, the client’s daughter was nearing the completion of her postgraduate studies and was preparing to get engaged to her boyfriend. As the client faced her daughter’s growing independence and distance, she experienced a brief episode of depression. Through therapy, she was able to identify her emotional state in a timely manner and received guidance on medical support, emotional regulation, and self-care strategies. By this point in therapy, a secure attachment had developed between the client and the therapist that gradually extended into her personal life, positively influencing her relationships with her daughter, husband, and siblings. The therapy sessions in this stage continued to monitor the client’s daughter’s binge-eating symptoms, and the client no longer felt excessive worry about her daughter’s eating habits. Instead, she developed greater trust in her daughter’s ability to manage herself. The client also began discussing plans for her later years with the therapist, exploring involvement in social charity work. She considered using her professional skills to contribute to the rehabilitation of individuals with mental health disorders. She also tried to think independently, make her own decisions, and return to a fulfilling life, striving to maintain a healthy family dynamic.

After Session 46, the therapist assigned the client a third narrative therapy homework titled “Us in Ten Years,” requiring the client to describe her family life as she envisioned it 10 years thence. This exercise applied the “enriching the new story” technique. In Session 47, the therapist and the client discussed the assignment. Through envisioning and reflecting on her future, the client realized that her daughter’s illness was only a phase in the family’s journey—it would be wrong to define her daughter by her disorder. While the illness had posed challenges to the family’s development, psychotherapy helped them overcome it. Although the healing process was difficult, it led to the client’s self-discovery, the formation of a healthier and more supportive mother-daughter relationship, and the restructuring of a previously dysfunctional family dynamic.

As the secure attachment fostered by the counseling relationship provided deeper nourishment to the client, her defenses gradually decreased, allowing her to explore the outside world more effectively and better perceive external people and events. She understood that her past excessive self-focus was a result of immature defenses. As she gradually relaxed internally and became less tense, she could also give more psychological space to her daughter, husband, and others.

##### 4.2.2.5 Stage five: termination and separation (sessions 51–54)

In the final session, the client bid farewell to the therapist in a calm and warm manner. By this point, the client’s daughter had completed her internship abroad and secured employment in the same city as her fiancé. Her life had become stable and fulfilling. The client had rekindled intimacy with her husband, stopped being overly critical of her siblings, and accepted that her daughter might not always respond promptly to messages. She learned to respect her daughter’s personal choices and autonomy. Additionally, she reduced her workload, allowing herself more time for quiet reflection and personal growth.

The therapist expressed joy and encouragement for the client’s progress and emphasized that the therapy process remained open-ended—she could always schedule additional sessions in the future if the client ever felt the need for further support.

The formation of a secure attachment in the relationship with her daughter involves being “present” in the relationship while maintaining healthy boundaries.

### 4.3 Reassessment and outcomes

At the end of therapy, Ms. D’s reassessment results were as follows: PHQ-9, 4 points (no significant depression symptoms), GAD-7: 3 points (no significant anxiety symptoms), PDQ-4: Paranoid Traits: 2.98, Narcissistic Traits: 5.12, Dependent Traits: 4.39, Passive-Aggressive Traits: 2.36. The evaluation indicated that her depression and anxiety had resolved, her paranoid thinking had improved, and her passive-aggressive relational patterns had diminished. However, some narcissistic and dependent traits remained, although at a milder level. Overall, as she completed therapy, Ms. D’s personality developed and her social function recovered.

As the therapy came to an end, the client was able to engage in a comprehensive self-reflection. She recognized that her neglect, belittling, resistance to attachment, and even instances of hitting and scolding had severely undermined her daughter’s psychological health during her daughter’s growth, especially in childhood. She saw that her past self was a deficient and cold mother. Through therapy, she gained insight into her past patterns and mistakes, acknowledged her own traumas and fears, and worked on healing her wounds from her early life and resolving her fears. The therapeutic process led to a transformation in the client, particularly as her attachment style shifted from insecure to secure. Consequently, she began to provide psychological support for her daughter, and her relationship with her husband also became harmonious and intimate. As she and her daughter grew and developed, the client was able to separate from her daughter while maintaining that connection, establishing healthy boundaries, and showing understanding and respect toward her daughter.

## 5 Discussion

### 5.1 Case reflection

From the biosocial theory perspective, emotional dysregulation in individuals with ED primarily stems from an “invalidating environment,” in which the individual’s experiences and emotions are often not acknowledged or accepted within the family setting. In such an environment, individuals frequently face rejection, criticism, punishment, or even abuse, leading to emotional neglect and the disruption of communication. Consequently, they develop maladaptive emotional expressions and dysfunctional coping mechanisms to manage anger. Families characterized by such dynamics often exhibit mismatches in temperament between parents and children, with affected children displaying heightened sensitivity, strong emotional reactivity, and slower recovery from distress. Prolonged exposure to such an environment contributes to severe emotional dysregulation, which may manifest as binge eating, food restriction, purging, self-harm, suicidal tendencies, impulsive spending, and substance dependence as means of alleviating or avoiding emotional distress.

Negative emotions can trigger both physical and psychological disorders. In family dynamics, the psychological maturity of parents significantly influences emotional enmeshment among family members. This enmeshment often results in low self-differentiation among children, leading to the direct transmission of parental anxiety, depression, worry, and shame. Consequently, the family emotional climate becomes oppressive, increasing the risk of physical and psychological disorders in children (13). Hence, providing psychological support to caregivers during the recovery process can be beneficial. Enhancing caregivers’ interpersonal trust, courage, self-affirmation, and emotional depth, along with appropriate psychoeducation and health education allows them to improve their emotional regulation and cognitive processing, in turn facilitating a higher level of mentalization, promoting changes in the core family emotional system, enhancing self-differentiation among family members, and fostering healthier family relationships (14). Psychological support is particularly effective due to its targeted nature—it can significantly improve caregivers’ mental well-being, enhance their self-efficacy, and alleviate their stress and psychological distress (15). Counseling and psychotherapy for caregivers play a crucial role in supporting patients with ED by providing emotional support to caregivers, helping them reshape their beliefs about the disorder, improving their quality of life, and increasing their awareness of their own struggles and reactions to the patient’s maladaptive behaviors (16). Counseling also serves as a medium for psychoeducation. A strong and secure therapeutic relationship enables clients to develop a sense of secure attachment, which can then be applied to real-life experiences, making them more adaptive and resilient.

In terms of the therapeutic mechanism, reviewing the overall treatment over 43 months, the healing factors are mainly reflected in the following aspects:

1. Accompanying exploration, through self-exploration, the client was also mirrored and contained, forming a clearer self-concept and reconstructing a secure attachment.
2. The treatment can be taken as an important part of the family support system, in the client’s individual therapy, the therapist and the client jointly faced the daughter ‘s recovery from BN, conducting effective psychological education and related work on illness management.
3. Ultimately helping the client form self-identity, these identities include mother, wife, businesswoman, etc., and reconstructing good self-consistency.

### 5.2 Methodology

In this case, the integration approach primarily involved technical eclecticism and assimilative integration. The technical eclecticism is reflected in the selection, application, or combination of specific therapeutic techniques and their timing and manner based on the needs of an individual client. In this case, the application of drawing and narrative therapies demonstrates the importance of using appropriate methods at the right time to facilitate therapeutic progress.

From the perspective of assimilative integration, the four major therapeutic approaches—cognitive-behavioral therapy, existential-humanistic therapy, psychodynamic therapy, and systemic therapy—continue to evolve and develop regardless of advances in approaches, as their approaches to client observation, logical thinking, experiential interpretation, and meaning construction extend throughout an individual’s lifespan (17). Based on these four paradigms, assimilative integration provides an innovative perspective of expansion and contributes to the creation and insight to improve the existing traditional thinking and practice. Assimilative integration allows therapists to expand the operating space while maintaining the original theoretical basis for clinical practitioners. In the process of assimilative integration, the Bowen family system therapy is the main line. The whole treatment process changes the triangular relationship of the client’s family, promotes the self-differentiation of the mother, regulates the emotional system and family projection process of the nuclear family, and cuts off the transmission of the mother’s negative emotions in the nuclear family, especially to the daughter. This process is also accompanied by cognitive changes and inner conflict and drive adjustment.

In this case, the psychotherapy process highlights that the core of integrative therapy is a focus on the client, which aligns with person-centered therapy (PCT) within the existential-humanistic approach proposed by Cooper and McLeod. Cooper suggests that the emphasis on “human value” in humanistic psychology influences psychotherapy through the following five ideas: ① A person is more than just the sum of their parts and cannot be reduced to their individual elements; ② a person’s existence is unique within their specific context and ecological environment; ③ a person has self-awareness and can reflect on their own awareness and surroundings; ④ a person has the ability to make choices and must take responsibility for them; and ⑤ a person has intentionality and is goal-directed, which can influence future events, leading them to seek meaning, value, and creativity. Empathy is built upon this understanding of human nature (18). The core philosophy of PCT involves conceptualizing the client holistically and establishing a deeply valued and respectful therapeutic relationship, focusing on the client’s potential and goals, and providing sufficient therapeutic conditions to help clients reconnect with reality, personal experiences, and value processing (19). Therefore, therapist empathy is essential in this process, extending beyond emotional engagement to include the cognitive understanding of clinical pathology, disease management, adolescent and geriatric psychological development, psychodynamic theory, and understanding of the client’s personal and family experiences. A comprehensive understanding of the client enables therapists to select the most appropriate psychotherapeutic techniques at the right time for effective intervention. In the current integrative therapy case, the family therapy component illustrates that by enhancing the emotional regulation and coping skills of Ms. D, the therapist helped improve her marital relationship, strengthened her family interactions, and facilitated her scientific understanding of EDs and her daughter’s personality development. This process empowered the mother, making her and the family a vital resource for the client’s recovery.

In general, psychotherapists’ competence includes three main dimensions: knowledge, skills, and attitude. The meta-competence of the psychotherapist is the foundation for the application of integrative-oriented therapy. The meta-competence paradigm of psychotherapy includes a therapist’s self-awareness, self-reflection, and self-assessment abilities. Under the meta-competence paradigm, therapists need to be aware of which techniques and knowledge are applicable in addressing clients’ issues, rather than being limited to a single school or technique; this involves the integration of a range of core techniques (20).

### 5.3 Mental illness management

Another important aspect in this case is the management of mental illness. During the process of working with the client (the mother of a BN patient), tracking and supervising the patient’s condition has always been part of the supportive work. Mental illness management is another aspect that the therapist needs to consider and be competent in.

Regarding mental health management, estimates from the World Health Organization indicate that over 25% of the global population experiences at least one form of mental or behavioral disorder in their lifetime (21). In China, data from the Chinese Center for Disease Control and Prevention (CDC) suggest that the number of individuals with mental illnesses exceeds 100 million, with a national prevalence rate of 15–20%. Among them, 16% suffer from severe mental illnesses, making mental health a significant public health and social concern (22). Social support theory posits that an individual’s mental and physical well-being is influenced not only by their personal cognitive and emotional resources but also by their social environment. Social support networks, including family members, peer groups, and close friends, play a vital role in helping individuals cope with life challenges. For individuals with mental disorders, timely and appropriate external support is particularly essential for symptom management and social reintegration. As primary caregivers for patients with ED, parents must familiarize themselves with key aspects of recovery, such as weight restoration, body image concerns, medical complications, medication management, the transition to flexible and independent eating habits, and the recovery of eating pleasure (23). Overall, mental illness management requires a multidisciplinary approach, including medication, behavioral training, and therapeutic interventions (24).

This case effectively demonstrates that psychotherapy and psychological care for caregivers of individuals with ED play an essential role in their recovery process. The involvement of caregivers’ psychotherapists in mental health management during the patient’s recovery process is also a critical element of family rehabilitation. Additionally, this case highlights the value and clinical applicability of the integrative therapy approach.

(Note: The client consented to publication of this case report).

## Data Availability

The original contributions presented in the study are included in the article/supplementary material. Further inquiries can be directed to the corresponding author.

## Footnotes

1 PHQ-9 Scale Standard: Score of 1–4, no clinical depression; 5–9, mild depression; 10–14, moderate depression; 15–19, moderate-severe depression; 20–27, severe depression.

2 GAD-7 Scale standard: Score of 0–4, no clinical anxiety; 5–9, mild anxiety; 10–14, moderate anxiety; ≥15, severe anxiety.

3 PDQ-4 factor standard: Paranoid Traits ≥ 4; Narcissistic Traits ≥ 5; Dependent Traits ≥ 5; Passive-Aggressive Traits ≥ 4.
